# Targeted Intraspinal Radiofrequency Ablation for Lumbar Spinal Stenosis

**DOI:** 10.7759/cureus.1090

**Published:** 2017-03-10

**Authors:** Robert E Jacobson, Michelle Granville, Jesse Hatgis, DO

**Affiliations:** 1 Miami Neurosurgical Center, University of Miami Hospital; 2 Pain Medicine, Larkin Community Hospital

**Keywords:** radiofrequency ablation, lumbar canal stenosis, lumbar facet cysts, epidural ligaments, posterior ligamentous complex, lumbar foraminal stenosis, neurogenic claudication

## Abstract

**Introduction:**

By using a combination of magnetic resonance imaging (MRI) and computed tomography (CT) of the lumbar spine, it is possible to distinguish between spinal stenosis caused by bone compression and specific soft tissue epidural intraspinal lesions that cause localized spinal canal stenosis and neural compression. Examples include facet cysts and yellow ligament hypertrophy. Many of these patients are elderly with medical comorbidities that make open surgery problematic.

**Materials & Methods:**

This is a study of patients with predominantly soft tissue stenosis being treated with targeted intraspinal radiofrequency (RF) heat ablation. This novel procedure is performed under local anesthesia in an outpatient setting using intra-operative imaging. Fine tip 20 gauge RF electrodes (Stryker® PA, USA) are precisely placed under radiologic guidance in the identified soft tissue causing the posterior compression of the lumbar spinal canal. After sensory and motor testing to make sure there is a safe distance of the needle tip from the nearby nerve roots to avoid any neural effect, multiple targeted lesions correlated by the MRI or CT scan are made in the fibrous and cystic soft tissue. Lesions are created using a focused 2 or 5 mm tip at 60 degrees centigrade (°C) for either 30 or 60 seconds. This heat causes sufficient shrinking of the targeted soft tissue resulting in relative reduction of the soft tissue component of the stenosis. This relative reduction in the stenosis of the spinal canal, similar to that measured with interspinous devices, provides long-term relief of symptoms, signs, and improvement of spinal motion in patients with lumbar stenosis. This report will review the spinal anatomy, and development and history of using RF in and around the nerve roots and epidural space, as it relates to lumbar stenosis. Examples of before and after MRI scans demonstrate the radiologic reduction in the size of the lesions. This soft tissue reduction correlates with patients' improvement in pain and clinical symptoms. Follow-up of the patients up to 30 months shows that the effect of RF heat on the soft tissue is long lasting.

**Results:**

In our long-term follow-up of greater than six months, 58% of RF treated patients had lasting relief of clinical symptoms, back pain, and claudication with increased spinal movement. This reduction in pain and improvement in motion allows patients to continue more aggressive physical therapy and muscle strengthening that secondarily can improve their symptoms. Post-procedure follow-up MRI scans in multiple patients have shown a clear reduction in soft tissue lesion size. Long-term follow-up demonstrated that 58% of patients treated with RF targeted ablation have not required further intervention and 22% went on to other surgical treatments for lumbar spinal stenosis.

**Conclusion:**

By reducing the soft tissue component of the stenosis with RF ablation and creating relatively more epidural space, targeted intraspinal RF may be a possible minimally invasive, percutaneous non-surgical alternative to treatment in a number of patients where soft tissue lumbar stenosis is the main cause of patients' symptoms. This technique offers a simple and safe additional method to relieve symptoms of lumbar stenosis and possibly compression within the neural foramina, especially in the elderly.

## Introduction

A subgroup of patients who failed conservative treatment for persistent pain and radiculopathy or neurogenic claudication due to radiologically documented lumbar stenosis were considered surgical candidates; however, due to age, associated medical conditions, or not wanting open surgery they were offered the possibility of using focused radiofrequency (RF) heat to reduce soft tissue elements of the stenosis. The epidural area to be lesioned was identified with computed tomography (CT) and magnetic resonance imaging (MRI) scans. Using intraoperative myelograms and/or CT guidance, under local anesthesia, one or more fine tip 2 or 5 mm RF electrodes (Stryker® PA, USA) were percutaneously placed in the specifically identified epidural target. Sensory and motor testing was performed to detect proximity to a nerve root or potential injury from RF current. If testing was negative, then sequential coagulating lesions were made. This report will demonstrate, both clinically and with direct comparison of pre- and post-lesioning MRI scans, how the RF cauterization causes shrinkage of the epidural tissue that was causing compression. The resultant reduction in soft tissue compression led to a relative improvement in the localized lumbar stenosis. Patient follow-up shows this procedure results in symptomatic relief lasting up to 30 months to date. Comparison of pre- and post-procedure MRI or CT scans after using RF shows progressive or persistent reduction in the size of the original compression, which correlated with continued symptomatic improvement. Eighty percent of patients had initially complete or greater than 50% relief of symptoms, and at six months follow-up was still maintained by 68% of patients. Long-term follow-up demonstrated that 58% of patients treated with RF-targeted ablation have not required further intervention and 22% went on to other surgical treatments for lumbar spinal stenosis. By reducing the soft tissue component of the stenosis via RF lesioning and creating relatively more space in the lumbar spinal canal, targeted intraspinal RF may be a possible minimally invasive, percutaneous non-surgical alternative to traditional treatment. This may apply to a number of patients where soft tissue lumbar stenosis is a part, or the major cause, of patients' symptoms and they either do not want open surgery or are poor surgical candidates.

## Materials and methods

1) Diagnostic studies including plain x-rays, CT, and MRI scans were reviewed in combination to identify the precise soft tissue causing foraminal and/or nerve root spinal stenosis. This can include large facet joint cysts and spinal stenosis due to marked ligamentous hypertrophy without significant bone stenosis. Importantly, many patients have a mixture of the aforementioned pathologies or some degree of bone hypertrophy, especially of the facet joints or associated degenerative spondylolisthesis.

2) Determine if the specific compression is percutaneously accessible through either the posterior spinal interlaminar space or laterally through the neural foramina. Even though many patients had multilevel lumbar pathology, the most severe one- or two-level of stenosis were the primary target.

3) The final patient selection was based on being symptomatic enough to be considered as an open surgical candidate; however, there were either sufficient medical contraindications to open surgery or the patient elected not to have open surgery. The patient was carefully explained the procedure, risks, and possibilities. The patient was cooperative enough to understand the following: the procedure would be done with local anesthesia and mild sedation, there would be intraoperative sensory and motor testing requiring his/her feedback after each stimulation, and an intraoperative contrast study would be performed to outline the target with myelogram dye, which is to be correlated with the location seen on CT and MRI.

4) The patient clearly understood that this was a new treatment option and he/she could still need an open surgery if symptoms persisted or worsened.

### Technique

**Figure 1:**
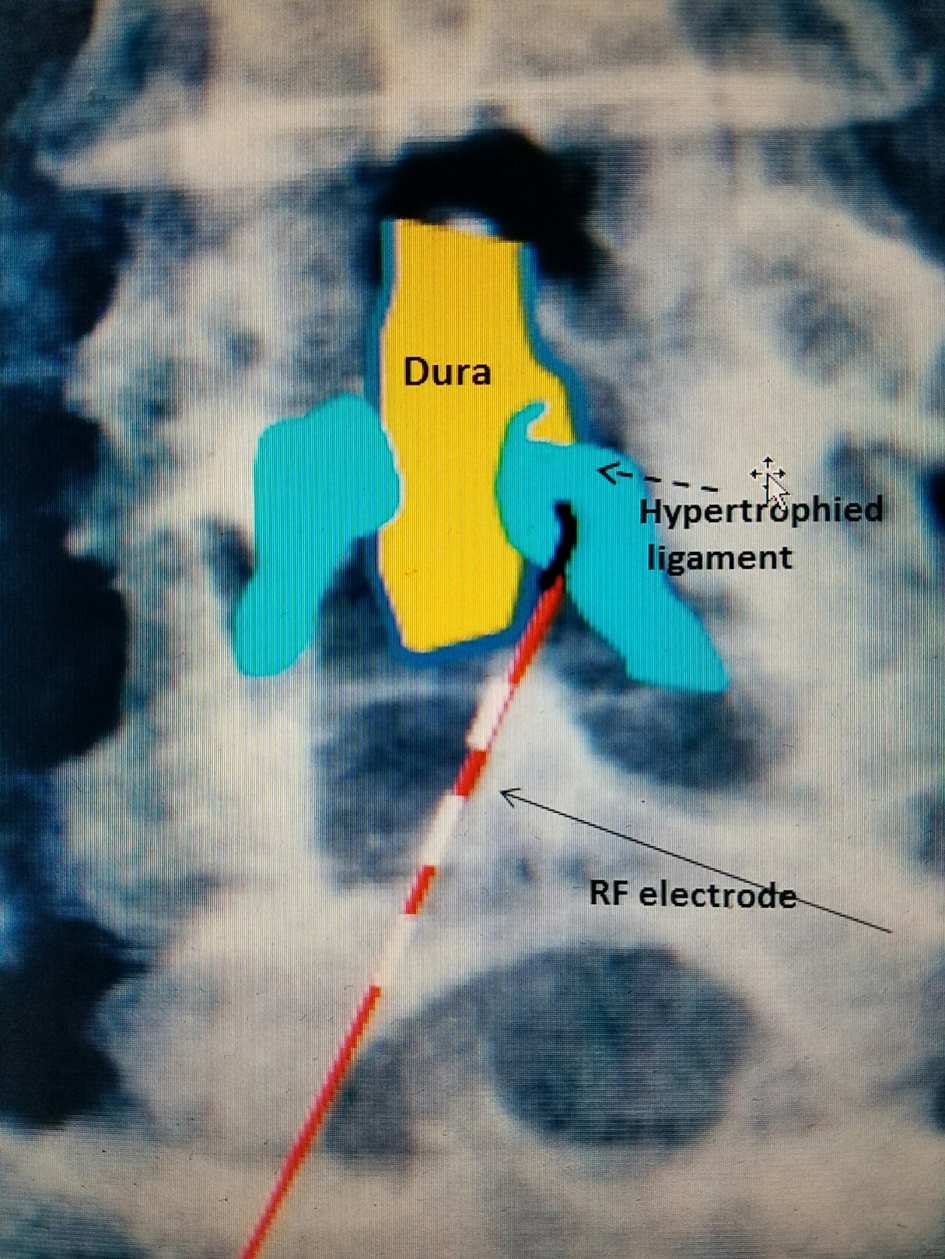
Anterior-posterior lumbar spine diagram 2 mm angled RF electrode (red with white dashes and black angled tip going underneath the lamina) entering hypertrophied yellow ligament (blue). RF: Radiofrequency.

**Figure 2:**
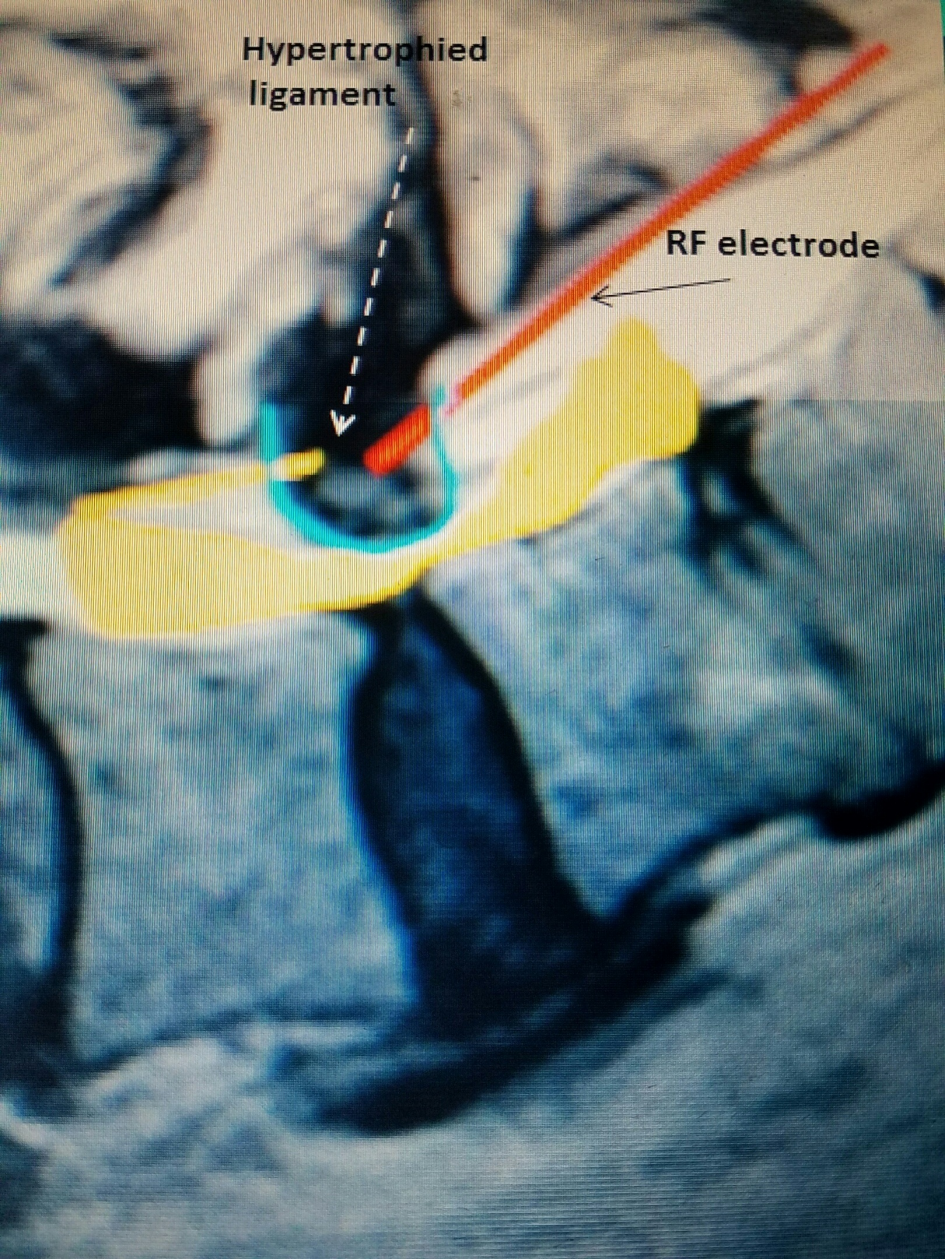
Lateral lumbar spine diagram Schematic of RF electrode (red line) entering the posterior epidural space into hypertrophied yellow ligament (represented by defect in the blue "U" and further noted by dashed arrow). RF: Radiofrequency.

The patient was given 1 g of cefazolin or 600 mg of clindamycin in the pre-holding area. In the procedure room, the patient was placed prone on the table and anterior-posterior (AP) fluoroscopy was used to identify alignment and any deformity of the lumbar spine. The skin area corresponding to the affected level and side was marked. After sterile preparation and draping, the skin was anesthetized with one percent lidocaine and a 22 gauge needle was used to perform a spinal tap, either above or below the targeted level. After clear cerebrospinal fluid (CSF) was obtained, 3 to 6 cc of water soluble contrast was injected into the subarachnoid space to perform a myelogram, outlining the target corresponding to the MRI/CT defect. Next, the skin was anesthetized with lidocaine at the location determined to allow the introduction of an electrode in the lumbar epidural space to approach the myelographic target. Then, a 15 or 20 cm 20 gauge RF electrode (Stryker®) with either an angled or straight 2 or 5 mm exposed tip was percutaneously placed at an oblique angle toward the intraspinal defect. (The angled trajectory was similar to that used when placing an epidural neuromodulator trial electrode into the posterior epidural space, but starting more laterally with advancement to the target on the contralateral side toward the lateral pathology.) The approach was either medial to lateral across the midline in the posterior epidural space, or inferior to superior into the yellow ligament through the interlaminar space if big enough when visualized under fluoroscopy. The target was the myelographic defect corresponding to the MRI/CT defect. The RF electrodes were 1.0 mm in diameter and 20 mm in length, with either 2 or 5 mm straight or slightly angled tips. AP, lateral, and oblique fluoroscopy was used to confirm electrode placement. The stylet was then withdrawn to confirm extradural tip placement and no CSF leakage (Please refer to Figures 1-2).

**Figure 3:**
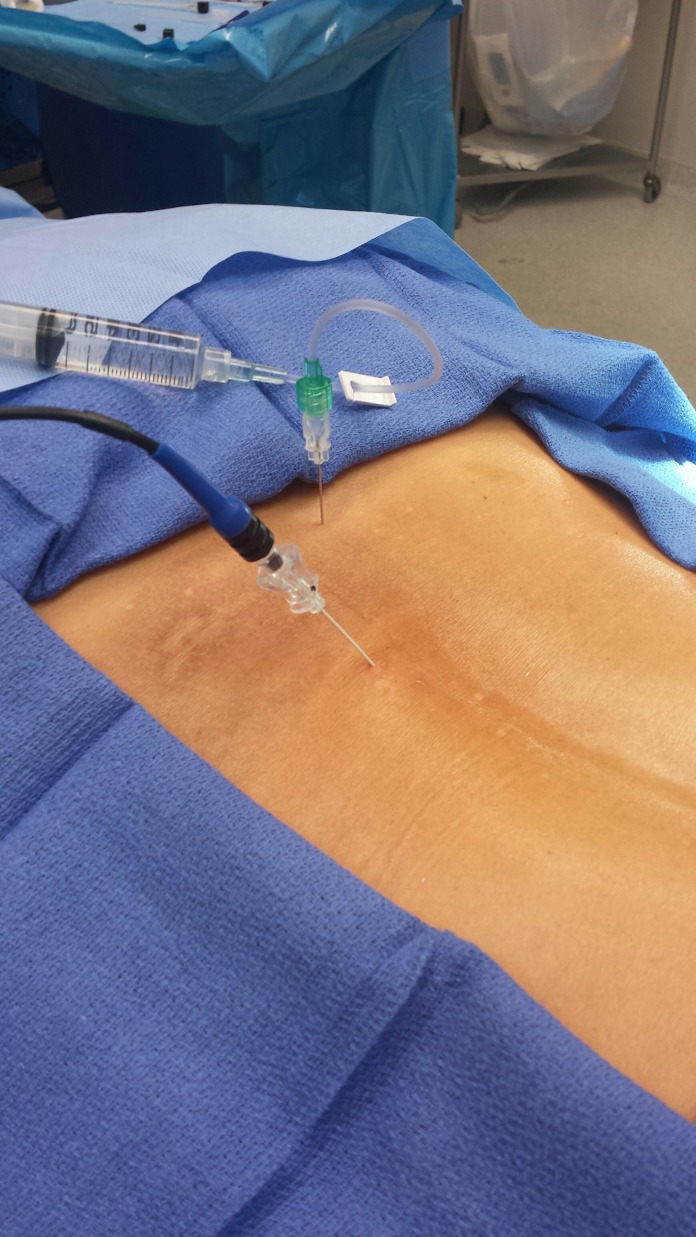
Patient with radiofrequency electrode in place RF electrode (blue hub needle) going into the epidural space. Contrast was used to outline the stenosis (green hub needle with attached tubing). RF: Radiofrequency.

The electrode was directed more laterally in the case of a facet cyst, which is usually anterior to the superior articular process. A transforaminal approach was used to target a facet cyst or lateral disc fragment. Electrodes could be placed unilaterally or bilaterally, and then sensory and motor testing of the electrode position was performed ranging from 0 mV to 2 mV. If persisting and/or increasing tingling sensation or twitching motion occurred, no lesion was made. If this occurred, the electrode was moved usually less than 1-2 mm and testing was repeated. When no sensation or movement occurred at maximum testing, then sequential lesions were made for 15, 30, and 60 seconds at 60°C. In cases with a large epidural lesion, several different RF electrodes were placed to cover the entire lesion. Again, sensory and motor testing was performed with each change in position and before each permanent lesion was generated, as shown in Figure 3.

After satisfactory lesioning, the electrode was removed, a small bandage was applied to the overlying skin, and the patient was returned to the recovery room. Final motor and sensory testing was performed on all patients prior to going to the recovery room and at the time of discharge from the outpatient center.

## Results

A total of 45 patients had the procedure performed over a 30-month period. Seventy percent were female and 30% were male.

Twenty-four patients had an MRI diagnosis of ligamentous hypertrophy with stenosis, 13 patients had grade 1 anterolisthesis at L4-5 of 5 mm or less, eight patients had multilevel spondylosis with predominant ligamentous stenosis at one level either at L2-3, L3-4, or L4-5. Twelve patients had unilateral facet cysts alone or in combination with stenosis or anterolisthesis. The patients were seen in follow-up within 7-14 days post-procedure. Subsequent visits were performed at six weeks, three months, and 12 months. The longest follow-up is 30 months. The follow-up of CT/or MRI was obtained in 18 patients with 11 patients showing the reduction in the targeted lesion.

Twenty-nine patients (64%) had 80+% post-procedure relief of subjective pain and claudication with the return of full spinal movement. Twenty-three patients (80%) of the group with initial relief had >6 months relief up to 30 months. For this subgroup, the average preoperative visual analog scale (VAS) score was 8, the average postoperative VAS at three months was 1-2, and at six months was 2. Regardless, the most favorable observation in this group was the marked improvement in spinal movement.

Seven of 45 patients (16%) had partial relief of leg complaints and improved walking with greater than 50% relief of residual complaints based on a preoperative VAS of 8 and postprocedure VAS of 3-4. Nine patients (20%) had no change in symptoms from the procedure: six of these nine patients elected to proceed with open surgery or placement of an interspinous/interlaminar device.

Patients who went on to have subsequent surgery for stenosis: post procedure follow-up showed that none of the 23 of 29 patients who had complete initial relief lasting six months or longer went on to surgery for stenosis in the 12-month follow-up (or beyond). Two of the six patients who did not maintain favorable results had later surgery for stenosis. Of the seven patients with partial relief (50%), three had later surgery. Five of nine patients with no change, or an aborted procedure due to inability of accessing the epidural space, underwent surgery for stenosis. In total, 10 of the original 45 patients (22%) underwent later surgery for stenosis. There were no detrimental effects or excess scarring found in any patient who had later surgery.

### Complications

1) Three cases had CSF exiting the RF cannula on initial placement. In the first case, the electrode was repositioned, lesioning continued and there was transient perineal numbness after the procedure. In the second case, a completely different electrode was repositioned without incident. In the third case, it was decided not to proceed with lesioning and the procedure was terminated because of the CSF leak and the cannula could not be repositioned.

2) There were no entry sites or intraspinal infections.

3) There were two patients with transient tingling in the nerve root adjacent to RF lesioning that persisted beyond 48 hours, but less than 96 hours. Both patients did not complain of radicular pain or continuous tingling at the time of RF lesioning during the procedure. There was no incident of motor weakness in either case. Both patients were given IV and oral corticosteroids with the resolution of their neurologic complaints.

4) Failed access: In two patients, bone obstruction or deformity prevented placement of the RF electrode into the myelographic or radiologic defect in the epidural space.

## Discussion

**Figure 4:**
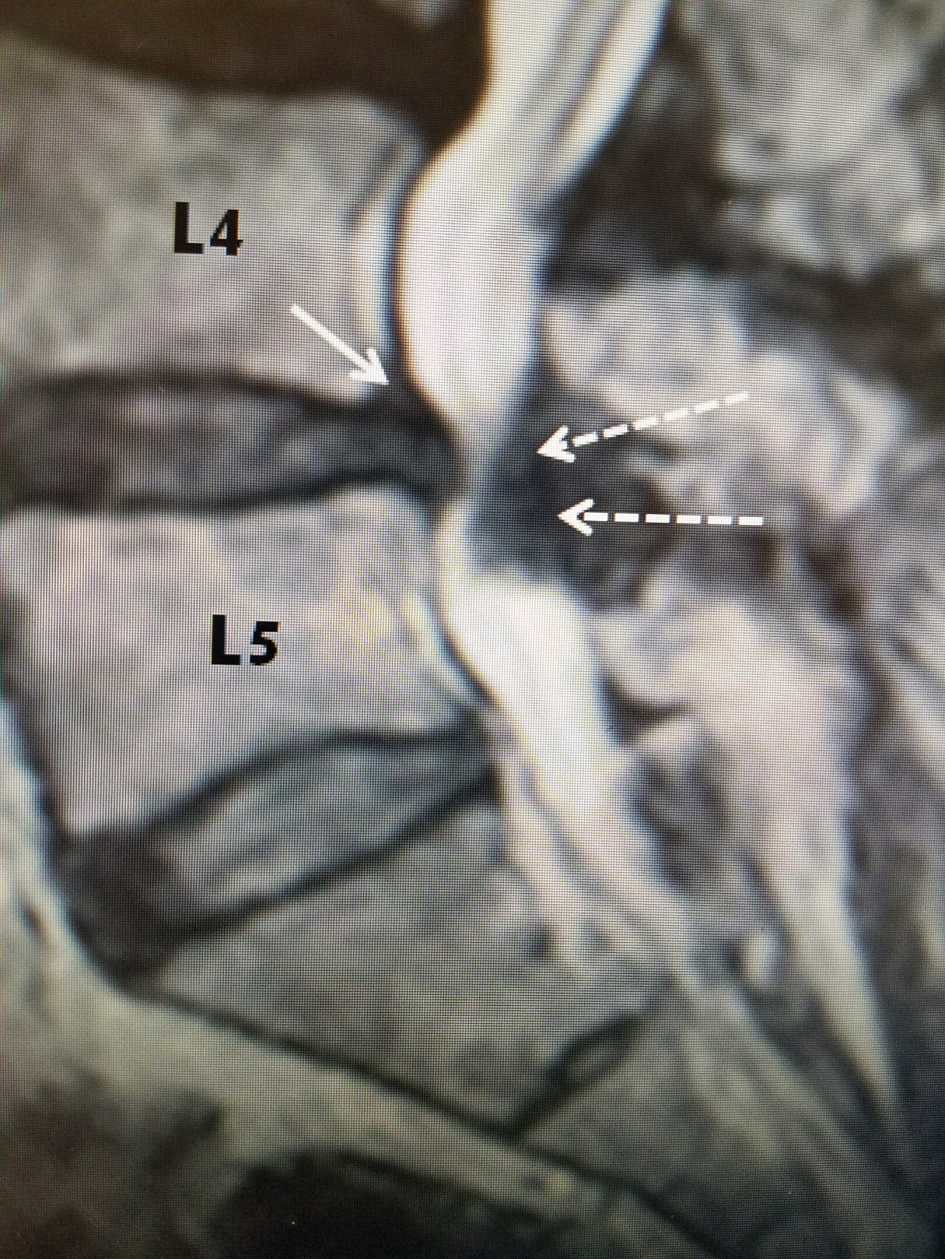
MRI of lumbar spine with L4-5 stenosis T2 sagittal MRI of L4-5 stenosis secondary to hypertrophied yellow ligament posteriorly (dashed white arrows) and minimal grade I L4-5 anterolisthesis with disc protrusion posteriorly (solid white arrow). Note that on T2 MRI, bone is a gray color and soft tissue, ligament, and disc annulus are darker. MRI: Magnetic resonance imaging.

**Figure 5:**
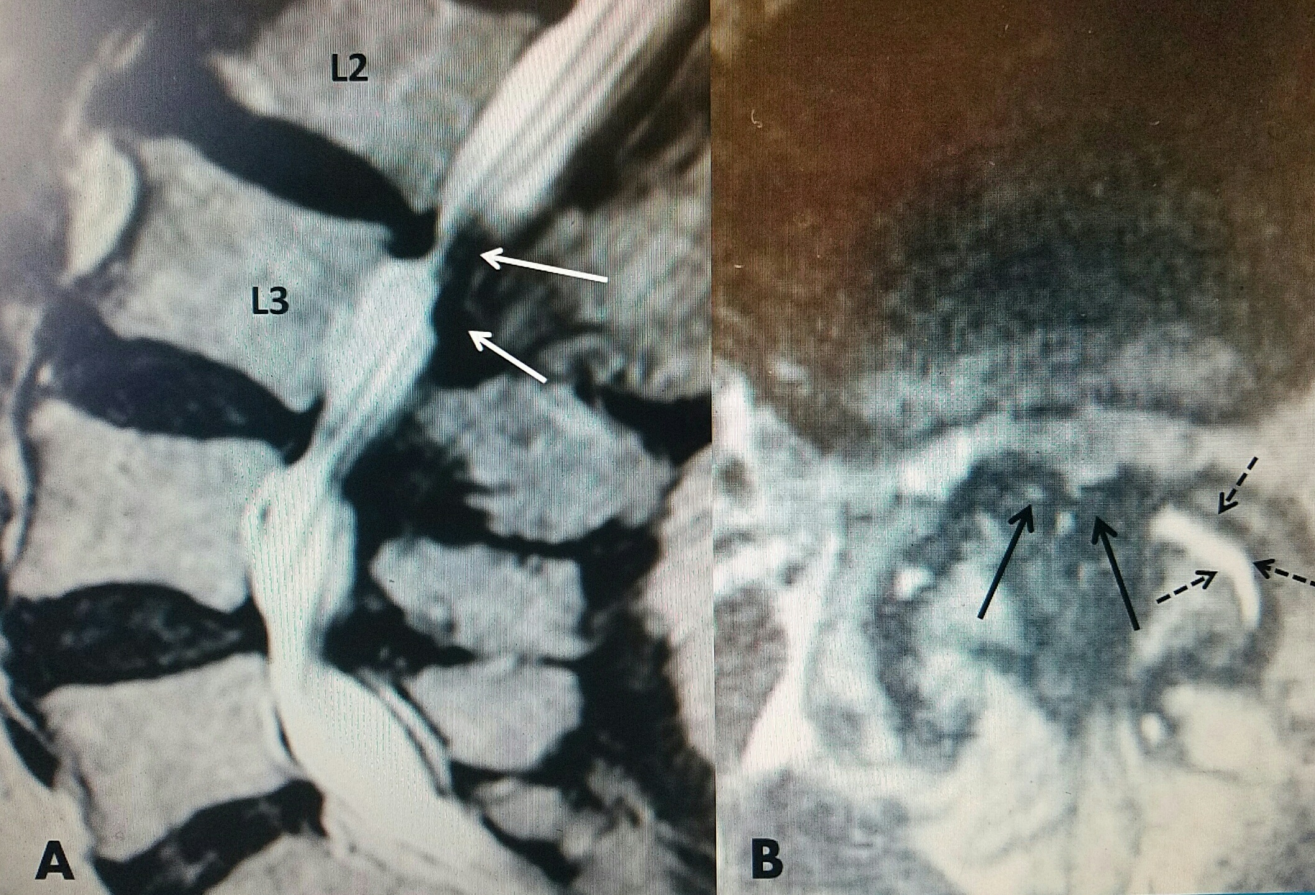
MRI showing multilevel stenosis with L2-3 ligamentous stenosis A: T2 sagittal MRI shows severe posterior L 2-3 ligamentous compression (white solid arrows) with a ventral L 2-3 disc. There is associated lesser degree of stenosis at L 3-4 and L 4-5. B: T2 axial MRI shows marked ligamentum flavum hypertrophy (black solid arrows) and marked fluid in the left facet joint (dashed arrows). MRI: Magnetic resonance imaging.

Spinal stenosis was first recognized by Verbiest in 1954, but was assumed to be primarily due to either a congenitally narrowed spinal canal or as a result of bone stenosis [1]. This was based on Verbiest’s measurements and observations primarily of bone specimens combined with intraoperative observations. In 1973, Epstein identified lateral recess stenosis as another cause of canal narrowing [2]. It was not until 1975 with the evolution of cross-sectional imaging and 1980's advances in CT and later MRI, that it became apparent that the soft tissue ligaments were a major cause of primary and also secondary stenosis, as shown in Figures 4-5 [3-6].

Currently, advances in spinal imaging using MRI, especially correlated with CT with reconstruction images, make it possible to clearly identify and separate compression secondary to bone verse soft tissue [7]. Foraminal and central canal compression due to bone and facet hypertrophy can be distinguished from that caused by herniated disc, migrated disc fragments, ligamentous hypertrophy, and spinal facet cysts [2-3]. Simultaneously, observations by numerous authors with many years of clinical experience evaluating and treating patients with lumbar stenosis clearly show that many elderly patients have developed clinically ‘silent’ stenosis that often is anatomically very severe yet these patient have minimal or no symptoms [6].  A subgroup developed clinical complaints that evolved only within a short time (typically months) before the radiographic studies demonstrated severe stenosis. It is common to see the symptoms and signs localized to one specific stenotic area rather than related to the more widespread or diffuse disease; however, patients are often explained that they need extensive multilevel decompression to address the more diffuse yet asymptomatic areas. More accurate MRI and CT imaging have also helped in the development of both percutaneous and open minimally invasive surgical procedures to remove the stenotic pathology. As a result, microsurgical decompression of the localized fragment, facet cyst, or ligamentous stenosis is possible [9]. Also, indirect canal decompression with posterior interlaminar devices without the surgical removal of bone or hypertrophied ligament demonstrate that moderate distraction provides up to 18% enlargement of the spinal canal area [10-11]. Preventing hyperextension often relieves many of the symptoms, which suggests that it may be sufficient to obtain more space without removing the pathology to get lasting clinical relief. Pedicle lengthening osteotomy was also suggested as a method of sagittally widening the stenotic canal and preliminary studies again demonstrate a relative enlargement of the axial lumbar canal after these procedures [12]. All of these more limited procedures attempt to create greater space to relieve root and cauda equina compression. Recent clinical reports consistently suggest that, especially in the elderly, it is sufficient to get limited axial widening either through interspinous devices or limited decompression. This gives satisfactory and lasting clinical relief in many patients, thereby avoiding more extensive surgery including multilevel decompressive laminectomy, foraminotomies with or without pedicle screw fixation [8-12]. In this report, we demonstrate that it is also possible, in specific cases, to get a similar result with targeted RF current that causes a reduction in the size of either the ligamentum flavum, facet capsule (FC) or a facet cyst that is the primary cause of the lumbar compression.

### Normal dimensions of the posterior lumbar epidural space and anatomic considerations

**Figure 6:**
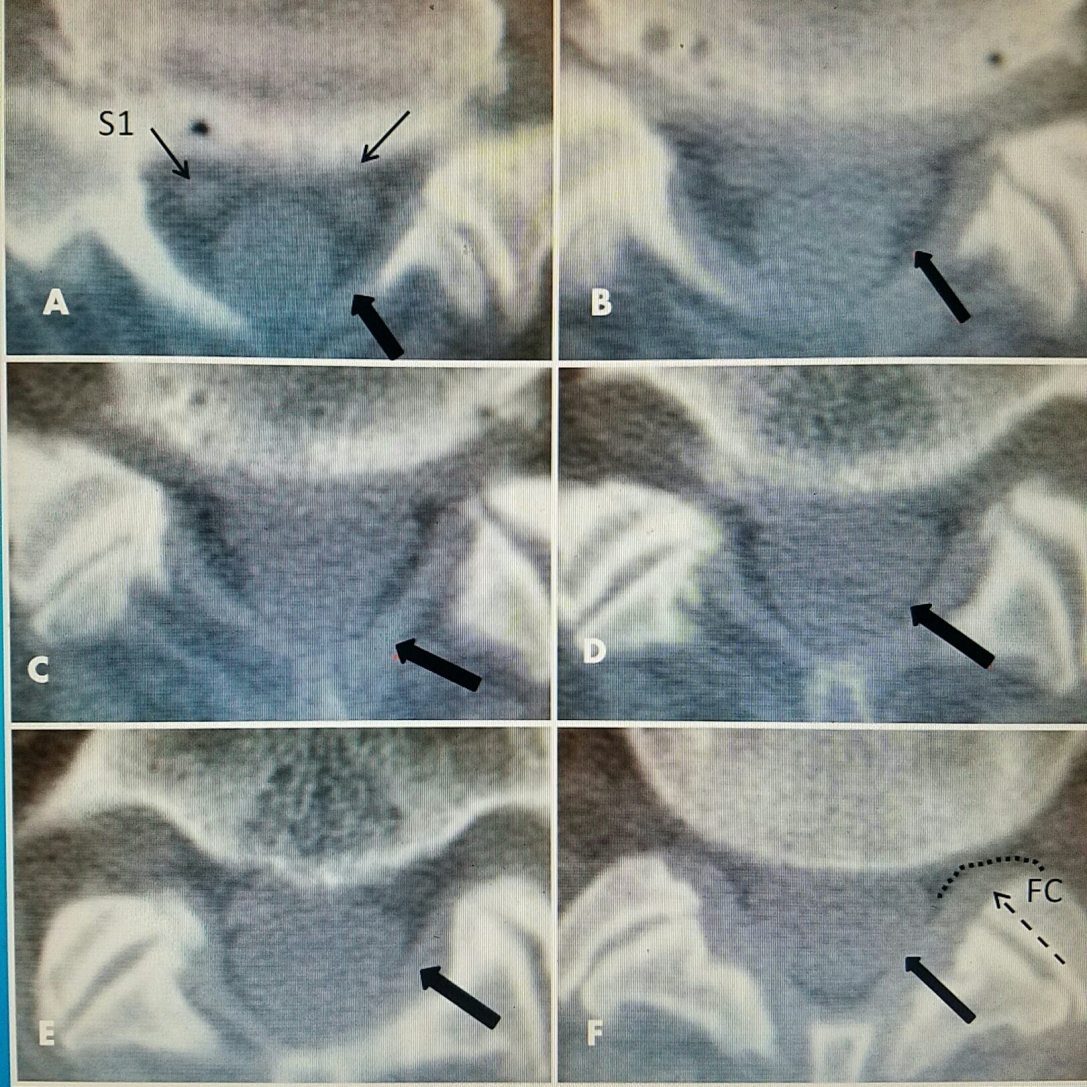
CT axial view of L5-S1 normal ligament flavum A-F: Clear delineation of ligamentum flavum (thick black arrows). A: S1 nerve roots in ventral floor of canal (thin black arrows). F: Facet capsule (FC) extending laterally at posterior part of neural foramina (dotted line and dashed arrow). CT: Computed tomography.

In studying axial CT or MRI images of the normal lumbar spinal canal, it is clear that the canal has a flat ventral base and an elevated laminar arch forming a triangle over the dura [3-4]. This creates a larger posterior epidural space between the dorsal laminar arch and the dura. The dura is positioned in the ventral part of the normal canal. The lamina extends laterally to become the inferior articular processes contributing to the formation of the facet joints, as shown in Figure 6 [2].

**Figure 7:**
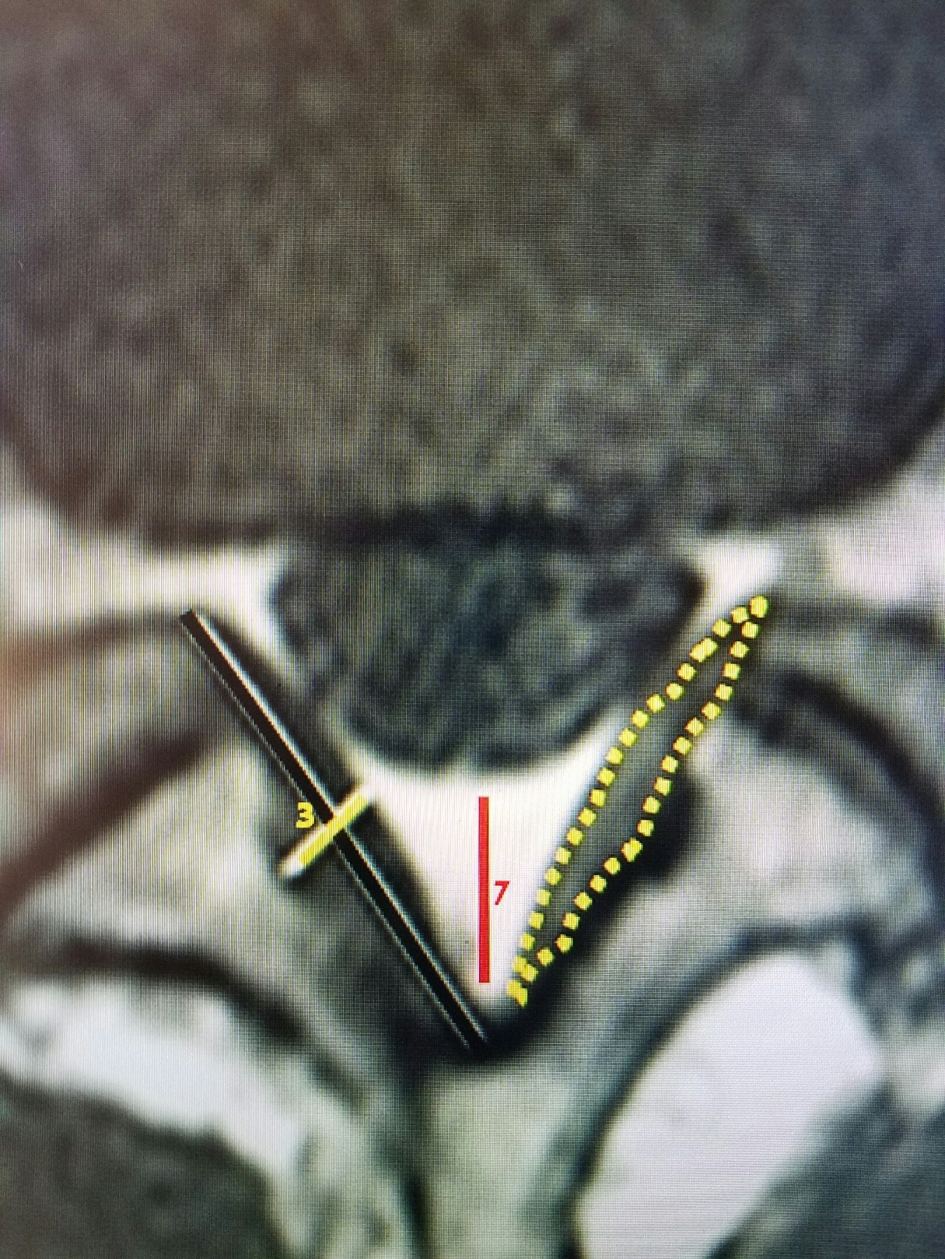
Axial view of normal L4-5 The red line shows normal epidural space, which varies from 4 to 7 mm at L4-5, dorsal to the thecal sac. The dotted yellow area outlines the ligamentum flavum. The straight yellow line shows the thickness of normal ligamentum flavum that is 2-3 mm in center.

Using MRI, the dimensions of the normal posterior epidural space can be documented, as shown in Figure 7. The distance from the posterior dural sac to the midpoint of the laminar arch is from 4-7 mm and normally filled with epidural fat. The normal ligamentum flavum attaches under the middle of the laminar arch of the superior vertebrae to the top ridge of the inferior arch of the inferior vertebrae. It never attaches medially. Moving laterally, the ligamentum flavum passes to the medial and ventral aspect of the inferior facet and the capsule of the facet joint. Measurements at its thickest in the midpoint are on average between 2.1 and 3.74 mm [3]. It becomes 0.1 to 0.3 mm thicker with advancing age (Figure 7). More laterally, it then forms the ligamentous capsule over the junction of the superior and inferior facet that is the ventral wall of the FC. This forms the posterior or dorsal wall of the foramina (Figure 6). The exiting nerve root and dorsal root ganglion pass anterior to the superior facet and yellow ligament.

### Pathologic anatomy

**Figure 8:**
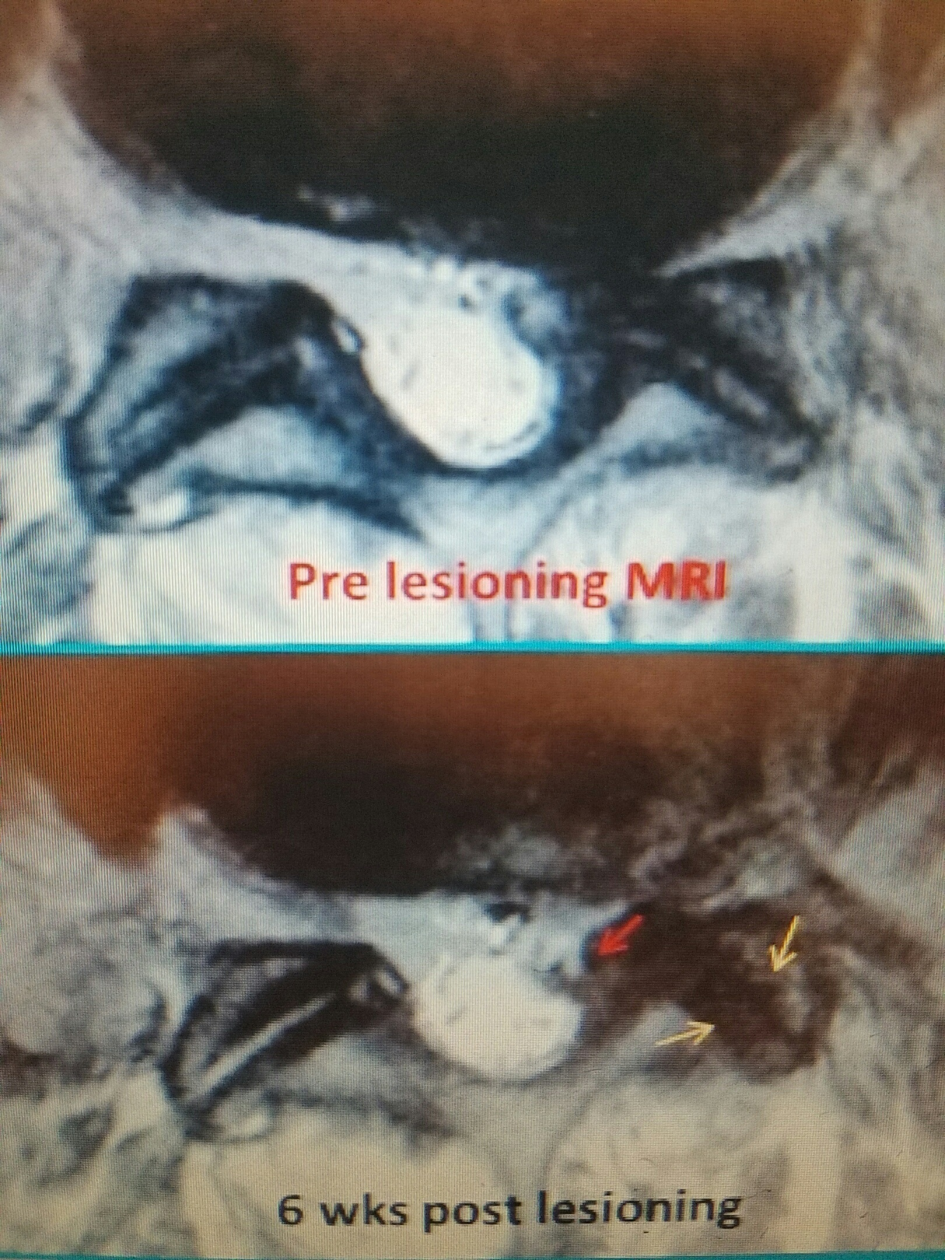
Pre- and postprocedure MRI of L5-S1 facet cyst 81-year-old with radicular pain from L5-S1 facet cyst. Pre-lesioning MRI shows left facet cyst with marked medial foraminal stenosis. Six-week postprocedure MRI shows soft part of cyst signifcantly reduced (red arrow). A small residual 'hook' spur off the medial edge of the inferior facet and less fluid in the facet joint (yellow arrows). MRI: Magnetic resonance imaging.

With ligamentous stenosis, the majority of soft tissue compression is caused by either unilateral or bilateral hypertrophy of the dorsal and lateral positioned yellow ligament, and with age, the ligament becomes thicker, looses elasticity and becomes composed of more collagen than elastin [2, 4, 7]. As the ligament enlarges, it fills the posterior and lateral epidural space that is normally filled with epidural fat and more laterally progressively narrows the foramina [2, 4] (Figure 8). Progressive thickening of the capsule and ligamentum flavum subsequently compresses the dura more medially and the nerve root laterally as it enters the neural foramina. Facet cysts are formed from a combination of hypertrophied capsule filled with fluid or mucoid substance from the hypertrophied facet joint. These cysts typically expand medially from the ventral capsule of the facet joint or into the spinal canal and neural foramina, which further compresses the foramina and epidural space [7-9]. This can then be reduced with RF lesions (Figure 8).

If there is associated disc herniation, disc degeneration, or anterolisthesis, then the space more ventral or the anterior border of the neural foramina is also narrowed. As the disc degenerates, it decreases in sagittal dimension. This leads to additional compression by a decrease in vertical height of the foramina between the pedicles above and below (Figure 7). As the space narrows, the superior facet of the lower vertebra, and part of the facet joint, forming the posterior wall of the foramina, ‘rides up’ creating narrower foramina and even more compression [2, 4, 8-9].

### Relationship of pathologic anatomy to placing of epidural RF electrodes

**Figure 9:**
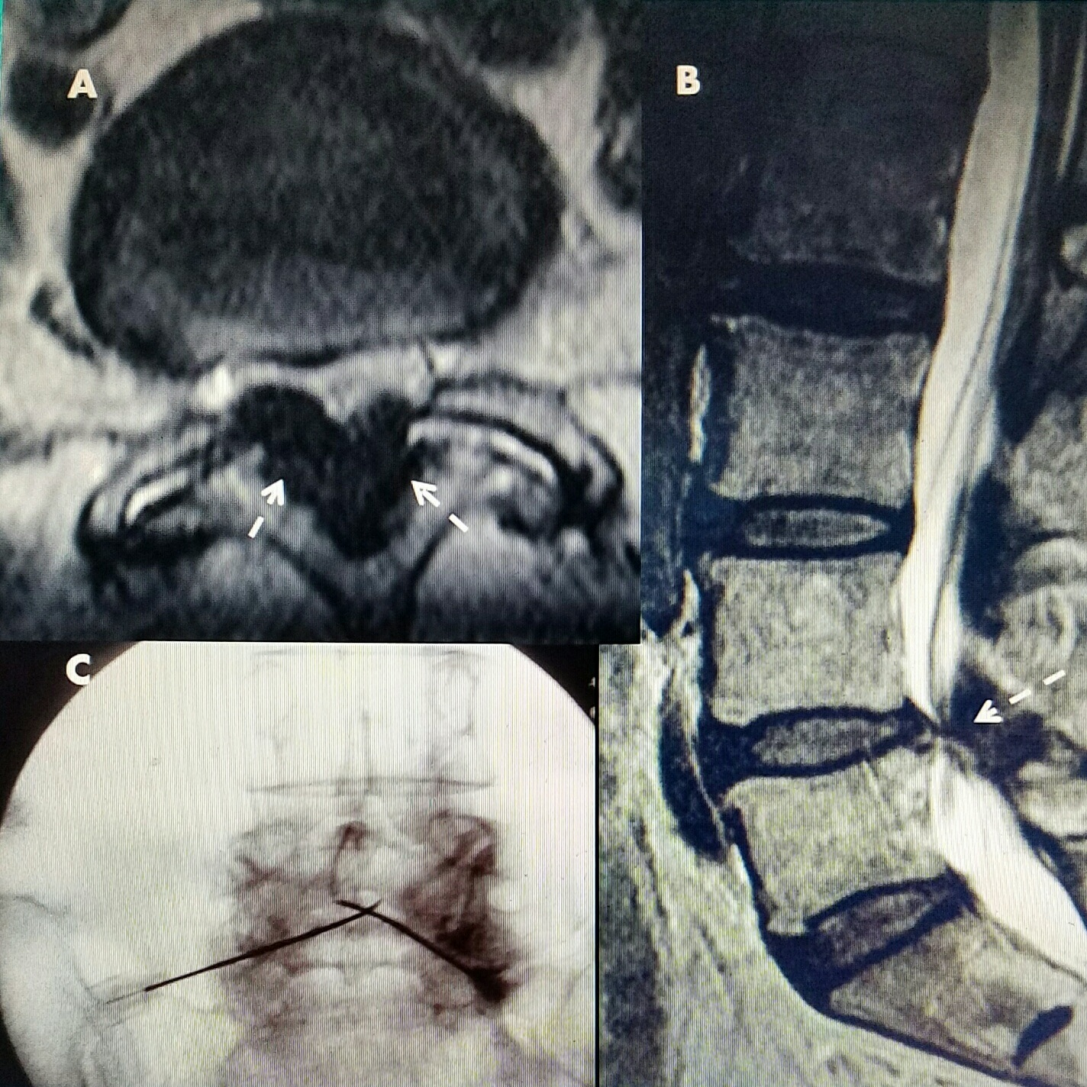
L4-5 stenosis radiofrequency electrode placement A: Axial CT showing marked ligamentous hypertrophy, (white arrows) which shows up hypointense and darker on T2 MRI. B: Sagittal T2 MRI with posterior compression from ligamentous stenosis at L4-5. C: Two RF electrodes placed percutaneously from inferior-laterally to superior-medially in posterior canal within ligamentum flavum. CT: Computed tomography; MRI: Magnetic resonance imaging; RF: Radiofrequency.

Since the hypertrophied ligament is always posterior to the dura, it is possible to place semi-rigid electrodes in this sublaminar position (Figure 9). The electrodes used are 0.8 to 1.0 mm in diameter (For comparison, an 18 gauge needle is 1.01 mm in diameter and an epidural neurostimulator electrode that is routinely used in the epidural space for spinal cord stimulation is 1.3 mm). By precisely guiding the RF electrode within the compressing lesion, the heat generated at the RF tip primarily affects the pathologic soft tissue for 2 to 3 mm [13]. Anatomically, it is not near or affecting the adjacent nerve roots. Pulsating CSF in the adjacent dura disperses the heat, avoiding intradural nerve injury (Figure 9).

### Merging use of RF lesioning with precise intraspinal targeting for lumbar stenosis

RF current has been used for very precise localized targeting in neurosurgery for over 50 years including lesioning of the trigeminal ganglion for trigeminal neuralgia at 60°C for 60 seconds, percutaneous RF cordotomy of the ascending cervical spinal thalamic tract for cancer pain at 70-85°C for 5 to 30 seconds, dorsal root entry zone lesions for cancer pain and RF hypophysectomy for metastatic tumor [13]. RF is also frequently used for ablating vertebral and other bone and liver tumors above 60°C [14-17]. It is also used for creating electro-silent lesions in the heart for cardiac arrhythmias [13]. All these RF systems use different arrays and sizes as well as angled electrode tips depending on the size and type of lesion being made. RF electrodes of various sizes are used often in the spine primarily for extraspinal lesioning of the facet joint capsule and the recurrent facet nerve for spinal pain control at 80°C for 90 seconds [14]. It is also used within the intervertebral disc and the disc capsule [14]. Multiple studies have been done showing the limit of heat spread and systems have been developed to simultaneously monitor the change in heat at different distances from the electrode tip.Technical studies of various RF electrodes show that the extent of the RF lesion is related to the original diameter and length of the exposed electrode tip as well as the temperature obtained [13, 15-16]. Detailed studies of the spread of current relative to the testing resistance of electrodes near the nerve root have been applied to RF treatment of vertebral lumbar osteomas and fractures [18-19]. Temperatures developed with RF can range from 45 to 80°C, which is just below the boiling point that coagulates the tissue in turn causing shrinkage of the soft tissue [13]. This leads to actual ‘ablation’ of the tissue similar to a laser which has been recently used for destruction of intraspinal epidural metastatic tumors [20].

**Figure 10:**
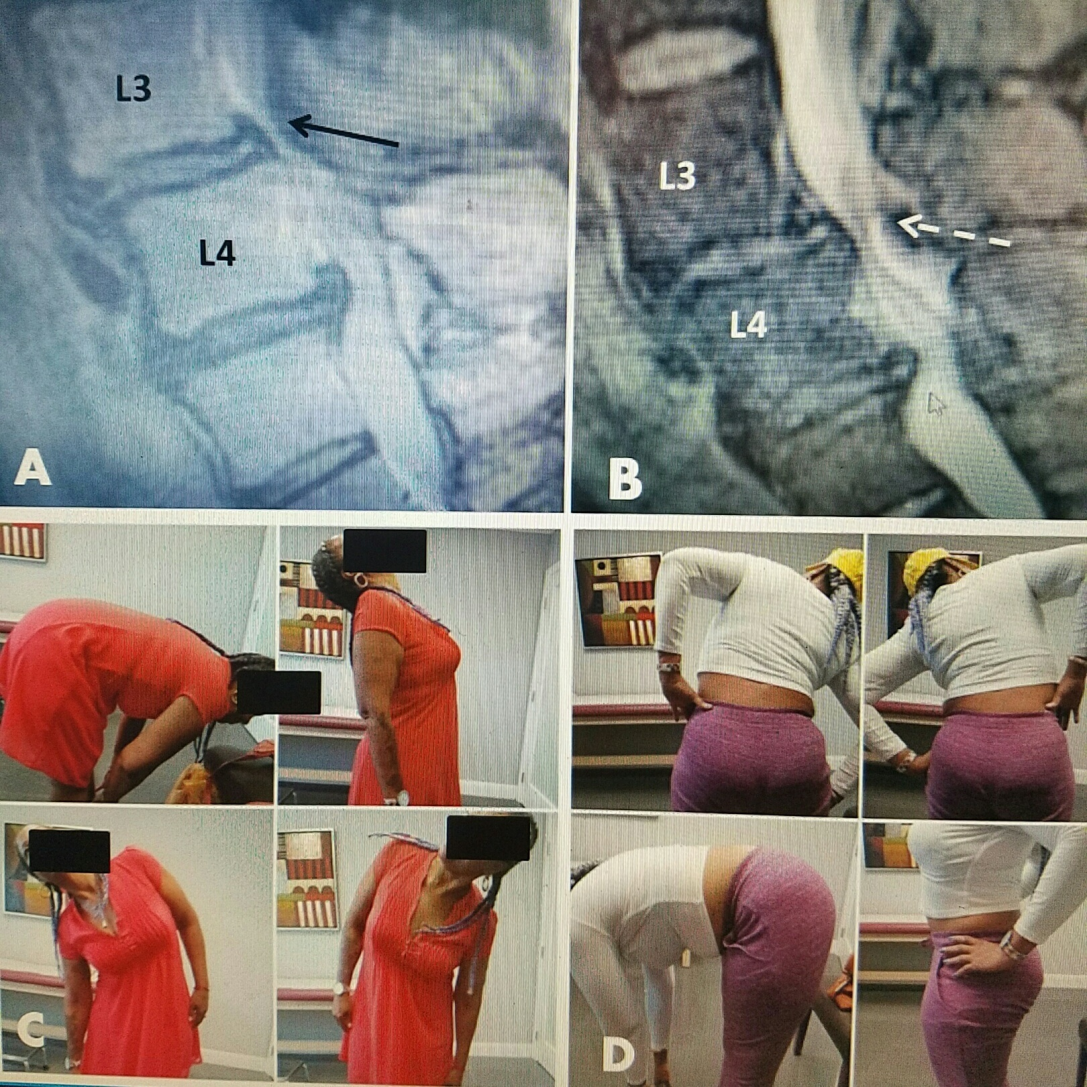
Patient movement after radiofrequency treatment for stenosis A: 58-year-old female with marked L3-4 stenosis (black arrow) with anterolisthesis at L3-4 and a small superior L4-5 disc extrusion. B: Post-treatment MRI follow-up at three months shows reduction in posterior ligamentous stenosis (white dashed arrow). C: Patient movement at three weeks post RF demonstrating full flexion, extension, and lateral bending. D: Patient continues with full normal movement six months post RF. MRI: Magnetic resonance imaging; RF: Radiofrequency.

Follow-up MRI and CT scans have demonstrated reduction in the soft tissue component of the stenosis at the targeted level with marked increases in patient movement, as shown in Figure 10.

The fact that only the most severe 1 or 2  levels of stenosis have been targeted, rather than attempting to treat multiple levels, with good results and lasting effect (up to 30 months) is supportive of the concept that many patients have multiple asymptomatic areas of stenosis, spondylolisthesis, and disc bulges but it is the relative stenosis that ultimately leads to the development of persistent symptoms and if this can be treated they can obtain lasting relief.

## Conclusions

This study demonstrates that it is technically feasible to safely make RF lesions in the soft tissue compressive pathology within the lumbar epidural space causing lumbar stenosis. If sufficient soft tissue is coagulated, patients can experience lasting clinical relief of their symptoms and improvement in spinal motion. As supported by other treatments such as microdecompression and interspinous or interlaminar spacers, it appears that in many patients with symptomatic lumbar stenosis, it is sufficient to create a relative increase in axial dimensions of the compressed lumbar stenosis to provide clinical symptomatic relief. In our long-term follow-up of greater than six months, 58% of RF-treated patients had lasting relief of clinical symptoms, back pain, and claudication with resulting increased spinal movement. This reduction in pain and improvement in motion allows patients to continue more aggressive physical therapy and muscle strengthening that secondarily can improve their symptoms. In this study, post-procedure follow-up MRI scans in multiple patients have shown a clear reduction in soft tissue compression. Out of the entire group, 22% went on to other surgical treatments for lumbar stenosis. Patients who went on to open surgery had no adverse effect or any abnormal scarring as a result of the previous RF procedure. Future improvement of this technique would include stereotactic and CT guidance as well as the design of better introducing cannulas to access the epidural space, and electrode tips that allow more modeling within the lesion. This has occurred with the use of RF in other areas of the body.
